# A study on the electron transport properties of ZnON semiconductors with respect to the relative anion content

**DOI:** 10.1038/srep24787

**Published:** 2016-04-21

**Authors:** Jozeph Park, Yang Soo Kim, Kyung-Chul Ok, Yun Chang Park, Hyun You Kim, Jin-Seong Park, Hyun-Suk Kim

**Affiliations:** 1 Department of Materials Science and Engineering, Korea Advanced Institute of Science and Technology, Daejeon 305-338, Republic of Korea; 2 Department of Materials Science and Engineering, Chungnam National University, Daejeon 305-764, Republic of Korea; 3 Division of Materials Science and Engineering, Hanyang University, Seoul 133-719, Republic of Korea; 4 National Nano Fab Center, Daejeon 305-806, Republic of Korea

## Abstract

High-mobility zinc oxynitride (ZnON) semiconductors were grown by RF sputtering using a Zn metal target in a plasma mixture of Ar, N_2_, and O_2_ gas. The RF power and the O_2_ to N_2_ gas flow rates were systematically adjusted to prepare a set of ZnON films with different relative anion contents. The carrier density was found to be greatly affected by the anion composition, while the electron mobility is determined by a fairly complex mechanism. First-principles calculations indicate that excess vacant nitrogen sites (V_N_) in N-rich ZnON disrupt the local electron conduction paths, which may be restored by having oxygen anions inserted therein. The latter are anticipated to enhance the electron mobility, and the exact process parameters that induce such a phenomenon can only be found experimentally. Contour plots of the Hall mobility and carrier density with respect to the RF power and O_2_ to N_2_ gas flow rate ratio indicate the existence of an optimum region where maximum electron mobility is obtained. Using ZnON films grown under the optimum conditions, the fabrication of high-performance devices with field-effect mobility values exceeding 120 cm^2^/Vs is demonstrated based on simple reactive RF sputtering methods.

The ever increasing demand for high-performance thin-film devices has led to the recent development of semiconductors with superior carrier transport properties^1^,^2^,^3^. Useful applications include high-resolution flat-panel displays, optical sensors, and multifunctional flexible electronics. While hydrogenated amorphous silicon (a-Si:H) remains the semiconductor of choice for thin-film transistors (TFTs) in the field of large-area electronics such as active-matrix liquid-crystal displays (LCDs) owing to the relatively low fabrication cost and sufficiently high areal uniformity^4^, alternative materials with mobility values exceeding 5 cm^2^/Vs have been extensively studied in an effort to realize more versatile field-effect devices. Note that a-Si:H TFTs exhibit field-effect mobility values no greater than 1 cm^2^/Vs, which is not sufficient for the realization of large-size (>100 inch), high-resolution (ultra-high-resolution, 4000 × 2000) flat-panel displays. A typical example of the research on high-mobility semiconductors is the work on two-dimensional metal dichalcogenides such as molybdenum disulfide (MoS_2_), which have attracted a great deal of interest thus far as pioneering materials in soft electronics^2^,^5^,^6^. However, appropriate synthesis methods at relatively low temperatures are not yet well established for such delicate materials; thus, many research groups strive to devise simple, practical layer-growth techniques^7^,^8^. On the other hand, another group of materials that exhibits excellent compatibility with current industrial thin-film processes is that of oxide semiconductors^9^,^10^,^11^,^12^,^13^, of which the most commonly used compound is indium-gallium-zinc oxide (In-Ga-Zn-O or IGZO)^1^,^14^,^15^.

The major advantage of oxide semiconductors is that they offer the possibility of sputter-depositing them at room temperature, resulting in layers with relatively high practical field-effect mobility values of approximately 10 cm^2^/Vs. The successful application of IGZO TFTs has allowed the mass production of high-resolution flat-panel displays of the types already commercially available. Reports in the literature indicate that such semiconductors are also suitable for the fabrication of mechanically flexible devices^16^,^17^. However, one of the major weaknesses of oxide semiconductor TFTs is their instability with respect to bias stress under illumination, which makes it difficult to guarantee a reasonable product lifetime^18^,^19^,^20^. It is generally reported that the presence of oxygen vacancies in oxide semiconductors affects the device reliability to a large extent, making it necessary to suppress the formation of oxygen-related defects in order to fabricate durable devices^21^,^22^,^23^. In this regard, a relatively novel type of semiconductor, specifically zinc oxynitride (ZnON), has lately enhanced the prospects for high-mobility devices that are stable with respect to bias stress under illumination^23^,^24^,^25^,^26^,^27^,^28^.

ZnON films may be grown by simple reactive sputtering using a Zn metal target in a mixture of oxygen and nitrogen gas plasma^23^,^24^. The implementation of ZnON semiconductors may thus be anticipated to be more cost-effective than the usual multi-cation compounds such as IGZO, which necessitate the use of relatively expensive raw materials such as indium or gallium. Although research has recently been carried out by several groups on the electrical performance and reliability of ZnON-based thin-film devices^23^,^24^,^25^,^26^,^27^,^28^,^29^, a proper understanding of the material properties in terms of their microstructure and chemical composition is not available in the literature thus far. The present work involves an extensive study of the influence of the deposition conditions on the physical and electronic properties of the resulting reactively sputtered ZnON layers. The sputtering power and the nitrogen/oxygen gas ratio are the main variable parameters during the growth process, and they influence the relative nitrogen to oxygen anion ratio. The crystal structure, microstructure, optical bandgap, chemical bonding state, and electrical properties of the resulting ZnON films are examined using X-ray diffraction (XRD), transmission electron microscopy (TEM), UV-vis spectrophotometry, X-ray photoemission spectrometry (XPS), and TFT current-voltage (I-V) measurements, respectively. It is shown that high plasma power and low oxygen gas flow rates lead to the formation of N-rich ZnON with a high electron carrier density level (>3 × 10^19^ cm^−3^). On the other hand, relatively weak plasma power and high oxygen gas flow rates result in the formation of films that are close to pure ZnO with low electron density (<5 × 10^18^ cm^−3^). The exact anion concentration cannot be determined with currently available characterization tools, and only approximate relative contents may be estimated using electron energy-loss spectroscopy (EELS) during TEM analyses. Density functional theory (DFT) calculations reveal that the incorporation of oxygen into a Zn_3_N_2_ host matrix may improve the material stability (the host has a highly negative oxygen affinity of −363.75 meV), and the formation of ZnON is thermodynamically more favorable, where oxygen acts as a mediator between the broken electron pathways in Zn_3_N_2_, thus enhancing the electron transport properties.

## Results and Discussion

### Hall Measurements

**Figure 1:**
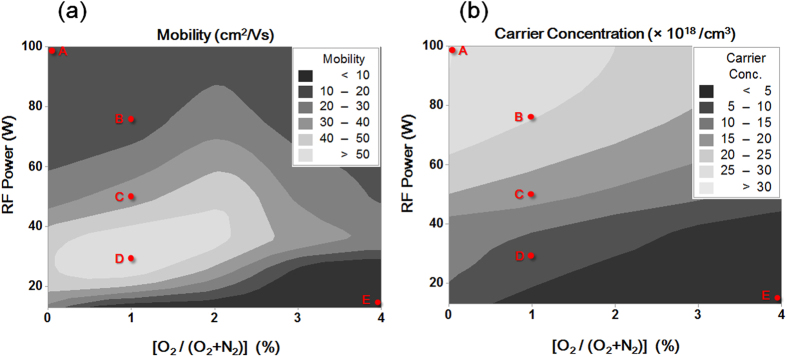
Contour plots of (a) Hall mobility and (b) carrier concentrations of ZnON films, with respect to the RF power and O_2_/(O_2_ + N_2_) ratio. Note that the carrier concentration exhibits a clear trend, while an optimum region exists for the electron mobility.

Thin films of ZnON were prepared with a thickness of approximately 50 nm, and their electrical properties were evaluated by Hall measurements with respect to the sputtering RF power and the oxygen flow rate ratio, *i.e*., O_2_/(O_2_ + N_2_). Figure 1(a,b) show a contour plot of the Hall mobility values and the electron concentrations. The mobility plot indicates that high electron mobility (>30 cm^2^/Vs) is obtained at relatively low oxygen gas flow rates (<2%) and between RF power levels of 20 and 40 W. The electron concentration plot shows that higher carrier densities are achieved at relatively high RF power levels and low oxygen flow rates.

While the effects of the sputtering power and oxygen flow rate result in a regular, predictable trend of the electron concentration, it is important to note that a region exists where the maximum Hall mobility is obtained. At relatively low power levels near 20 W, the mobility values decrease considerably to below 10 cm^2^/Vs when oxygen the gas flow rates exceed 2%. On the other hand, moderately high mobility levels between 10 and 40 cm^2^/Vs are obtained at all oxygen flow rates with RF powers above 40 W.

Such different behavior with respect to the film growth parameters is anticipated to be related to the microstructure, chemical composition and bonding properties that determine the carrier transport mechanism. To perform the necessary characterizations, specimens were prepared on bare glass under the conditions indicated in Fig. 1(a,b), as A (100 W, 0%), B (75 W, 1%), C (50 W, 1%), D (30 W, 1%) and E (15 W, 4%).

### Microstructure

**Figure 2:**
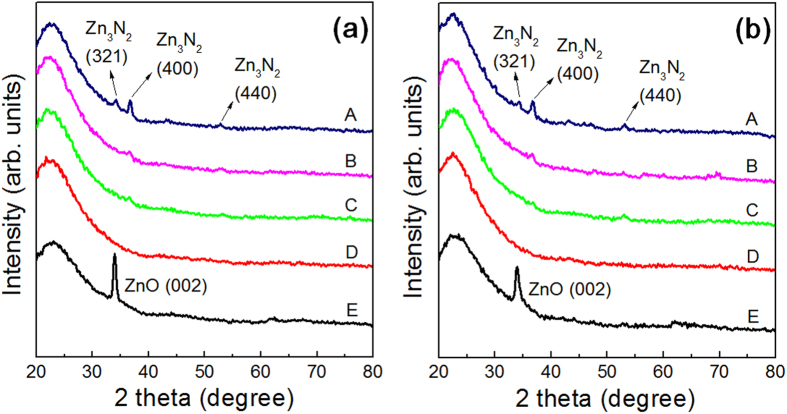
Grazing incidence angle X-ray diffraction (GIAXRD) patterns of films A through E, in the (a) as-deposited state and (b) after annealing at 250 °C for 1 hr in air. Peaks that correspond to those of cubic Zn_3_N_2_ appear in the N-rich film A, while films B, C, and D exhibit structure that are close to being amorphous. Film E consists of crystalline ZnO with a strong (002) peak.

Grazing incidence angle X-ray diffraction (GIAXRD) was used to investigate the crystallinity of the films with sufficient X-ray intensity in order to obtain as much information as possible from the nanocrystalline features, as shown in Fig. 2(a,b). The microstructures of the as-deposited films (Fig. 2(a)) and films annealed at 250 °C for 1 hr (Fig. 2(b)) appear to be identical. Film A exhibits peaks that correspond to those of polycrystalline zinc nitride (Zn_3_N_2_), showing a strong (400) peak. The peaks intensities decrease considerably in the case of film B, which implies that a substantial fraction of the crystalline Zn_3_N_2_ phase becomes amorphous. Further lowering of the sputtering power results in XRD profiles that appear to be closer to those of amorphous layers (films C and D); however, previous studies indicate that such ZnON films may consist of nanocrystallites embedded in an amorphous matrix^26^. Therefore, more accurate analyses by high-resolution TEM must be carried out. For film E, a clear ZnO (002) peak is observed, indicating the presence of ZnO crystallites grown in the [002] direction. In conjunction with the Hall measurement results, it may be stated that the presence of grain boundaries in crystalline films (A and E) impedes the motion of free carriers and decreases the mobility as a result. The films that exhibit minute crystalline portions (films B, C and D) also show relatively high electron mobility values, a result that is most likely due to the absence of grain boundary scattering effects.

**Figure 3:**
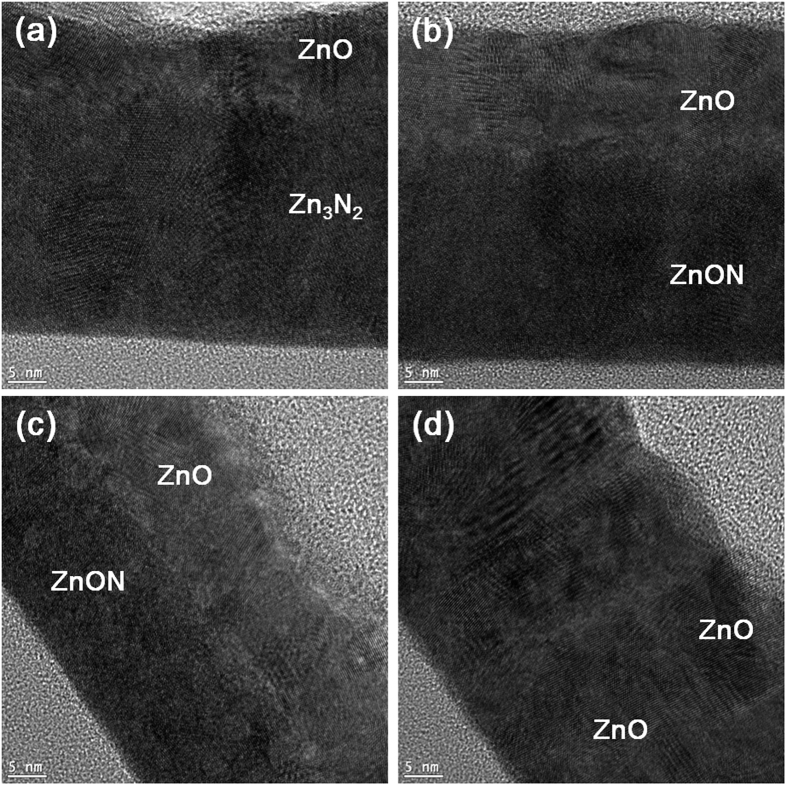
High magnification TEM images of films (a) A, (b) B, (c) D and (d) E. A clear polycrystalline phase is observed in film A, while a mixture of nanocrystallites embedded in an amorphous matrix is observed for film B. Film D exhibits a structure that is rather close to being completely amorphous, and film E consists of columnar ZnO.

**Figure 4:**
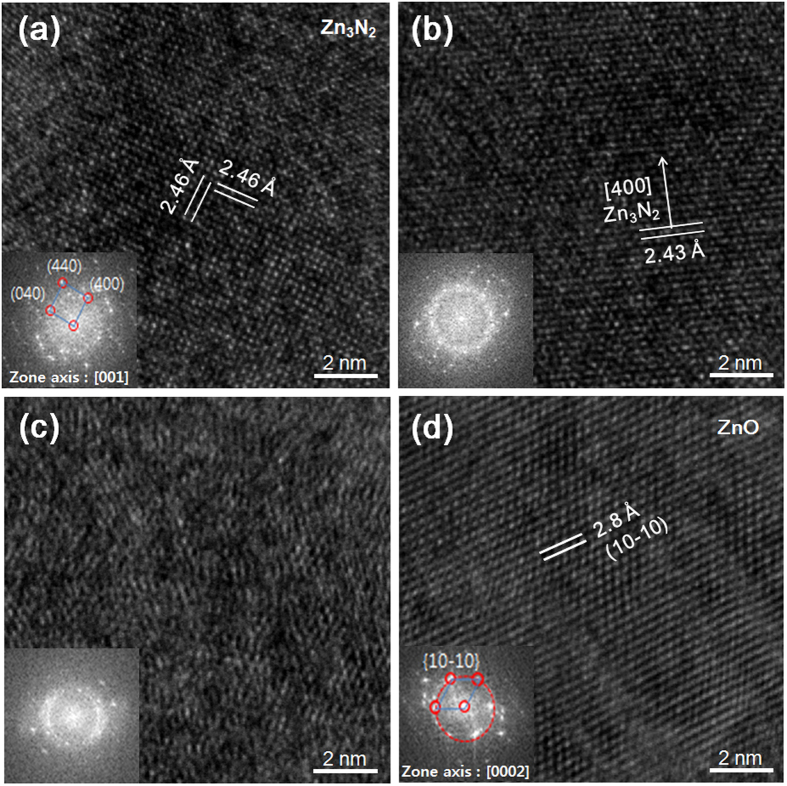
High resolution TEM images of films (a) A, (b) B, (c) D and (d) E. A clear cubic Zn_3_N_2_ structure is seen along the [001] zone axis for film A in (**a**). The inset consists of a fast Fourier transform (FFT) of the observed area, confirming the presence of a cubic phase. Film B consists of a cubic Zn_3_N_2_ nanocrystallite that could be identified within an amorphous ZnON matrix in (**b**). Film D consists mostly of an amorphous matrix, in which it is difficult to identify exactly which phase is present in the regions that appear to exhibit some crystallinity as shown in (**c**). Film E consists of columnar ZnO, of which a grain is identified in (**d**).

High-resolution TEM images are shown in Fig. 3(a) through (d) of films A, B, D and E, respectively (See the Supplementary Information for low magnification TEM images). In order to avoid oxidation of the films upon prolonged exposure to air^31^, thin capping layers of ZnO approximately 10~15 nm thick were sputter-deposited on top of each film. A clear polycrystalline phase is observed in film A (Fig. 3(a)), while a mixture of nanocrystallites embedded in an amorphous matrix is observed for film B (Fig. 3(b)). Film D exhibits a structure that is fairly close to being completely amorphous (Fig. 3(c)), and film E consists of columnar ZnO (Fig. 3(d)). Further magnifications are shown in Fig. 4(a–d). A clear cubic Zn_3_N_2_ structure is seen along the [001] zone axis for film A (Fig. 4(a)). The inset shows the fast Fourier transform (FFT) of the observed area, confirming the presence of a cubic phase. Figure 4(b) shows a cubic Zn_3_N_2_ nanocrystallite that could be identified within an amorphous ZnON matrix. In the case of film D shown in Fig. 4(c), most of the film consists of an amorphous matrix, and it is difficult to identify exactly which phase is present in the regions that appear to exhibit some crystallinity. One may thus refer to film D as a quasi-amorphous ZnON structure. Finally, for film E, a clear ZnO grain is identified (Fig. 4(d)), and amorphous regions are scarcely noticed.

### Chemical Composition and Bonding States

**Figure 5:**
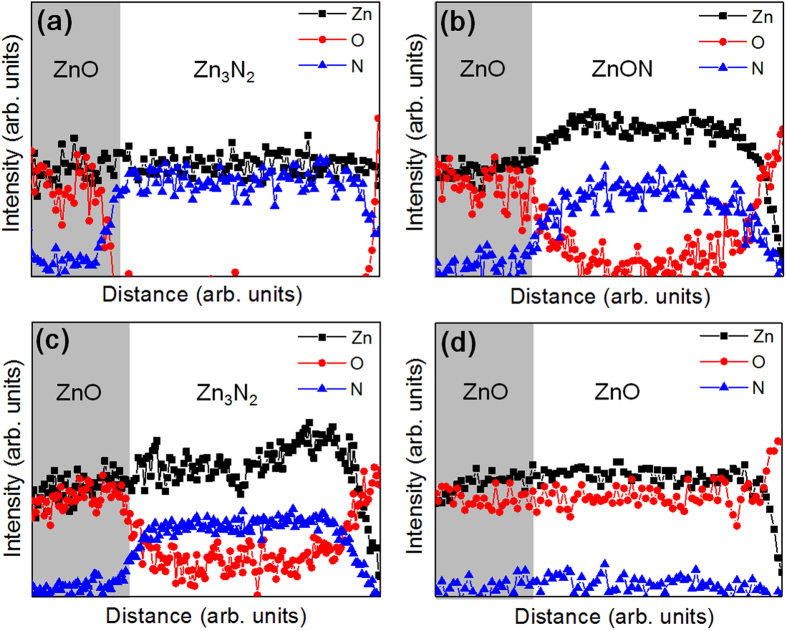
Line profiles of electron energy loss spectroscopy (EELS) spectra, for films (a) A, (b) B, (c) D and (d) E. Line scan profiles starting from the top capping ZnO layer indicate that film A contains only nitrogen cations indicated in (**a**), of which the concentration decreases with increasing O_2_ flow rate, ultimately becoming negligible in film E (pure ZnO) as shown in (**d**). The relative concentration of oxygen anions increases accordingly from film A to E.

The chemical compositions of the films were measured by electron energy-loss spectroscopy (EELS), and the line profiles are shown in Fig. 5(a) through (d) for films A, B, D, and E, respectively. Line scan profiles starting from the top capping ZnO layer indicate that indeed film A contains only nitrogen anions, of which the concentration decreases with an increase in the O_2_ flow rate, ultimately becoming negligible in film E (pure ZnO). The relative concentration of oxygen anions increases accordingly from film A to E. The overall Zn cation concentration remains approximately constant, while only the relative concentration of oxygen to nitrogen is modified because two competitive reactions take place during the film growth process, one tending to form Zn_3_N_2_ and the other forming ZnO^24^.

**Figure 6:**
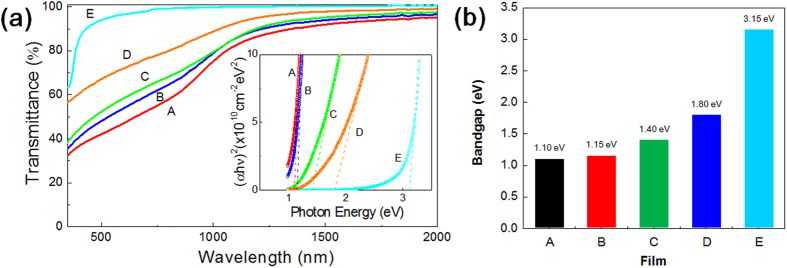
(**a**) Optical transmittance of films A through E measured with a UV-Vis spectrophotometer. (**b**) Optical bandgap values of films A through E extracted using the Tauc method. The bandgap values of film A and E correspond to those of pure zinc nitride and zinc oxide, respectively, and films B, C and D exhibit intermediate bandgap values between those of films A and B.

The optical transmittance of each film was measured, as shown in Fig. 6(a), and the optical bandgap values were extracted using the Tauc method^30^, as listed in Fig. 6(b). Note that the bandgap values of films A and E correspond to those of pure zinc nitride and zinc oxide, respectively, while films B, C and D exhibit intermediate bandgap values between those of films A and E.

**Figure 7:**
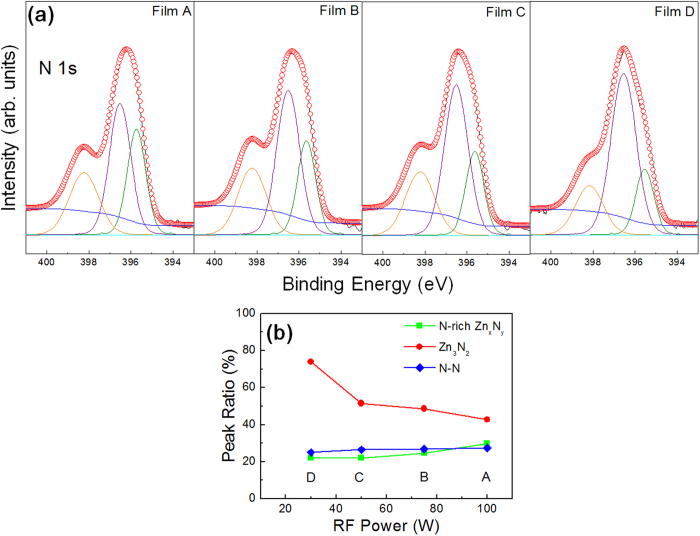
(**a**) X-ray photoemission spectroscopy (XPS) analyses on films A through D. The nitrogen 1 s peaks were observed for each film, while film E was excluded since it does not contain any detectable level of nitrogen. The N 1 s peaks were deconvoluted into three different sub-peaks, each located at 395.8 eV, 396.5 eV, and 398.3 eV. The lowest energy sub-peak at 395.8 eV originates from nitrogen atoms in N-rich zinc nitride, while the middle sub-peak at 396.5 eV arises from the nitrogen atoms in stoichiometric Zn_3_N_2_. The highest energy sub-peak at 398.3 eV represents mostly N-N bonds. (**b**) The relative intensity ratios of the sub-peaks in films A through D.

X-ray photoemission spectroscopy (XPS) analyses were also carried out in order to examine the chemical bonding states by observing the nitrogen 1 s peaks, as shown in Fig. 7(a), for films A, B, C, and D. Film E was excluded, as its nitrogen content is below the detection limit of the XPS instrument. The N 1s peaks were deconvoluted into three different sub-peaks, located at 395.8 eV, 396.5 eV, and 398.3 eV^28^,^32^,^33^,^34^. The lowest energy sub-peak at 395.8 eV originates from nitrogen atoms in N-rich zinc nitride, while the middle sub-peak at 396.5 eV arises from the nitrogen atoms in stoichiometric Zn_3_N_2_. The highest energy sub-peak at 398.3 eV represents mostly N-N bonds. The relative intensity ratios of the three sub-peaks within a single N 1s peak are represented in Fig. 7 (b). Note that as the RF power decreases, the contribution of N atoms in stoichiometric Zn_3_N_2_ increases, which suggests that the ZnON films deposited at relatively low power levels contain a larger fraction of stoichiometric Zn_3_N_2_ bonds, while the layers grown at higher power levels contain excess nitrogen.

The above results lead us to suspect that the bonding state may be strongly correlated with the carrier mobility of the ZnON films. The absolute nitrogen content increases with an increase in the plasma power and a decrease in the oxygen flow rates during growth, and the same trend is observed for the carrier concentration values, such that N-rich layers (with a composition close to Zn_3_N_2_) contain free electrons at higher densities. Nitrogen vacancies (V_N_) are generally known to act as shallow donors in nitrides^35^,^36^,^37^, and N-rich films are therefore expected to contain larger numbers of V_N_ defects, resulting in relatively high free-carrier concentrations. On the other hand, the Hall mobility variations do not precisely match the anion compositions and the carrier concentration. For this reason, it is conjectured that a more complex mechanism governing the carrier transport in ZnON may exist. The XPS results provide preliminary insight into the relationship between the electron mobility and the relative number of stoichiometric Zn_3_N_2_ bonds in the material. For instance, film D exhibits the highest electron mobility among the layers under consideration. Excluding pure ZnO (film E), film D contain the greatest number of oxygen anions while exhibiting the highest fraction of nitrogen anions forming stoichiometric bonds with a Zn_3_N_2_ configuration. In this regard, density functional theory (DFT) calculations were undertaken in order to elucidate how oxygen incorporation in the nitride system may influence the characteristics of the material.

### Density Functional Theory (DFT) Calculations

To describe the electron distribution in the Zn_3_N_2_ and ZnON matrices accurately, the HSE06 hybrid exchange-correlation functional^38^ was employed for all calculations, with 32% of the HF exchange energy modified to fit the experimental bandgap of Zn_3_N_2_ (DFT-calculated bandgap: 1.06 eV, experimental bandgap: 1.06 eV)^39^. Later, the V_N_ was filled with an oxygen atom to examine the effect of incorporating oxygen into the formation of ZnON. Of particular interest is the substitution of V_N_ with oxygen. The thermodynamic oxygen affinity per Zn ion of non-stoichiometric Zn_3_N_2_ (with a V_N_ in the unit cell) was calculated as follows:


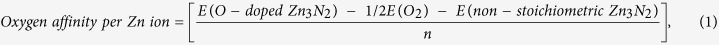


where *E*(O-doped Zn_3_N_2_), 1/2*E*(O_2_), and *E*(non-stoichiometric Zn_3_N_2_) represent the DFT-calculated total energy levels of the corresponding systems and where n denotes the number of Zn ions in the primitive cell of O-doped Zn_3_N_2_, which is typically 24. The calculated oxygen affinity of non-stoichiometric Zn_3_N_2_ is −363.75 meV. Note that this value can vary with respect to the local environment of the oxygen anion within the Zn_3_N_2_ matrix. The negative oxygen affinity of non-stoichiometric Zn_3_N_2_ confirms that the formation of ZnON is thermodynamically favorable.

**Figure 8:**
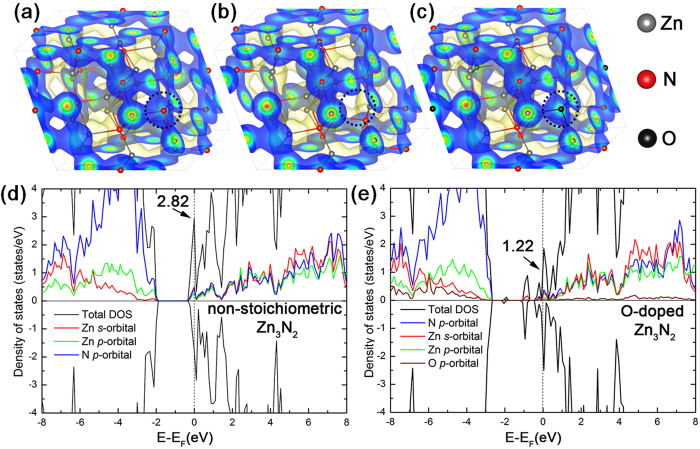
Cell structures and electronic properties of the Zn_3_N_2_ systems under consideration. A primitive unit cell of (**a**) stoichiometric Zn_3_N_2_, (**b**) non-stoichiometric Zn_3_N_2_ (with a nitrogen vacant site, V_N_), and (**c**) O-doped Zn_3_N_2_ (with oxygen replacing the V_N_ site). The yellow filled area represents the iso-surface with an electron density of 0.04 e/Å^3^. The density of states (DOS) of non-stoichiometric Zn_3_N_2_ and O-doped Zn_3_N_2_ are presented in (**d**,**e**), respectively. The dotted vertical lines denote the locations of the Fermi level in each case.

Figure 8(a–c) show the overall electron density distributions of the following three systems: stoichiometric Zn_3_N_2_, non-stoichiometric Zn_3_N_2_, and O-doped Zn_3_N_2_. Notably, the V_N_ breaks the charge continuity of the Zn_3_N_2_ matrix and locally disconnects the pathway for facile electron conduction (Fig. 8(a,b)). The oxygen atom that fills in the V_N_ reconnects the broken electron network of the non-stoichiometric Zn_3_N_2_ matrix (Fig. 8(c)). This result corresponds to the experimentally observed mobility enhancement with the addition of oxygen (film A → B → C → D), as indicated in Fig. 1(a).

Figure 8(d,e) show the density of states (DOS) of non-stoichiometric Zn_3_N_2_ and O-doped Zn_3_N_2_ (refer to Fig. 8(b,c) for the geometries). The DOS of non-stoichiometric Zn_3_N_2_ at the Fermi level is 231% (2.82/1.22) larger than that of O-doped Zn_3_N_2_ such that relatively low free-carrier densities are expected in the latter. This result is consistent with the experimental observations presented in Fig. 1(b), confirming that the incorporation of oxygen in zinc nitride decreases the carrier concentration.

The overall effects of the incorporation of oxygen into Zn_3_N_2_ based on the DFT calculations are: 1) mobility enhancement by the local restoration of electron pathways, and 2) the suppression of electron carriers (supporting the experimental findings).

### Thin-Film Transistor (TFT) Properties

**Figure 9:**
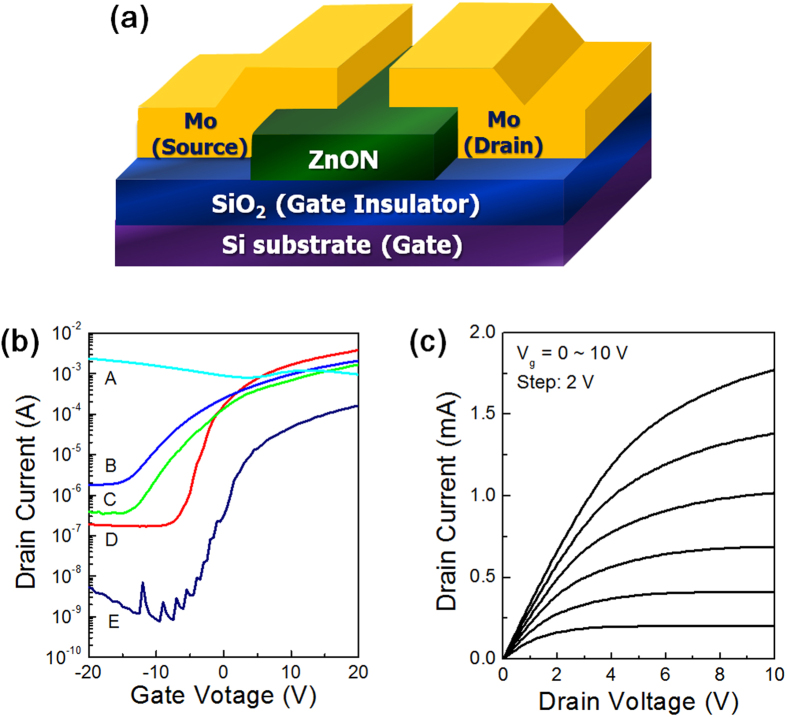
(**a**) Schematic of the fabricated ZnON TFT device with an inverted staggered bottom-gate structure. (**b**) Transfer curves (I_d_-V_g_) acquired with a V_d_ of 10 V for devices A through E. (**c**) Output (I_d_-V_d_) curves of device D, collected with different V_g_ values (0~10 V).

The different values of the Hall mobility and electron density in ZnON determine the corresponding TFT device performance. Figure 9(a) is a schematic illustration of bottom-gate ZnON TFTs fabricated with molybdenum (Mo) source-drain top contact electrodes. Such a structure does not necessitate complicated fabrication procedures such as photolithography and does not alter the surface of ZnON. Therefore, straightforward information pertaining to the field-effect properties of the semiconductor layer is provided with this configuration. The transfer characteristics of devices A through E, each involving the use of ZnON films A, B, C, D and E, respectively, are shown in Fig. 9(b). The drain current (I_d_) values on logarithmic scale are plotted against the gate voltage (V_g_) values on a linear scale. The output curves of device D, reflecting the drain current (I_d_) versus the drain voltage (V_d_), are shown in Fig. 9(c). The field-effect mobility (μ_FE_) values along with the threshold voltage (V_th_) and subthreshold swing (S.S.) values were extracted in compliance with the gradual channel approximation^40^, as listed in Table 1. Note that the same trend in the field-effect mobility is observed compared to that obtained by the Hall measurements of each individual film. Film A contains an excessively high carrier concentration, making it impossible to deplete the channel region of the associated device with the gate voltage. Film A can thus be regarded as a highly conductive metal-like material. As the carrier concentration decreases, devices B through E behave as switching elements, in which the carrier density in the channel can be modulated by the gate voltage. Relatively low absolute values of the gate voltage are required in order to turn the devices on and off with low carrier densities, which enables the fabrication of economical electronics in terms of power consumption. The absolute value of the threshold voltage decreases from device B to device E, suitably reflecting that the possibility of modulating the channel with the applied gate bias is enhanced when the carrier concentration is kept below a certain range (<3 × 10^19^ cm^−3^), as shown in Fig. 1(b). While the field-effect mobility of devices B and C are fairly elevated, device D exhibits excellent electrical performance, with its field-effect mobility exceeding 120 cm^2^/Vs. Such a high mobility level is seldom observed in simple TFTs fabricated with conventional sputtering techniques^41^. The output curves show that saturation occurs at relatively high V_d_ values, implying that film D exhibits indeed semiconducting properties.

Here, the control of the carrier mobility and concentration in the initial ZnON layers directly influences the TFT properties. First, it is best to keep the carrier concentration as low as possible to obtain reasonable V_th_ values in the resulting device. Next, the reactive sputter process must be optimized in order to identify which condition provides the highest electron conduction path. This can be done by considering the Hall mobility of the ZnON films, for example by considering a contour plot such as that shown in Fig. 1(a). A balance between the Hall mobility and carrier density can thus be established, which can then be transferred to the corresponding TFT device.

## Conclusions

The reactive sputter synthesis of ZnON films and their electrical properties were studied with respect to the process parameters, thin-film microstructure and chemical composition. From the results obtained in this study, it may be concluded that the electrical properties of ZnON films can be controlled by tuning the growth conditions. While the carrier concentration can be determined by the relative amount of nitrogen content in the layers, DFT calculations indicate that the electron mobility follows quite a complex mechanism involving the interaction between vacant nitrogen sites and oxygen anions incorporated during growth. The high-conductivity electron pathways that are locally disconnected in the vicinity of a nitrogen vacancy (V_N_) are suggested to be restored by the incorporation of an oxygen anion therein, which results in high Hall mobility layers and TFT devices with high field-effect mobility levels.

High-performance devices with field-effect mobility exceeding 120 cm^2^/Vs could be fabricated by properly adjusting the process parameters. The latter conditions result in the synthesis of high Hall mobility ZnON films, with relatively low carrier concentrations such that the associated TFTs exhibit high field-effect mobility and reasonable V_th_ values within applicable ranges. Such high-performance devices pave the way towards the realization of future high-resolution large area displays, system-on-glass (SOG), or system-on-plastic (SOP) platforms.

## Methods

### Experimental Section

Thin films of Zn-O-N were deposited by reactive radio-frequency (RF) magnetron sputtering using a Zn metal target in a mixture of argon (Ar), oxygen (O_2_) and nitrogen (N_2_) plasma. A TLP1005 smart sputter system by Teraleader Corporation was used for the deposition of all Zn-O-N layers. 50-nm-thick films were deposited onto SiO_2_/Si wafers and glass substrates at room temperature. The film stoichiometry was controlled during the deposition step by adjusting the oxygen flow rate ratio, O_2_/(O_2_ + N_2_), from 0 to 4%, and the RF sputtering power from 15 to100 W. The working pressure (6.5 mTorr) and Ar flow rate (5 sccm) were kept constant. In order to prevent oxidation of the ZnON films upon prolonged exposure to air prior to the thin-film characterization process, either thin capping layers (10~15 nm) of ZnO were deposited onto the ZnON films or post-annealing was done in air at 250 °C for 1 hr to oxidize the surface slightly.

The crystal structures of the films were examined by grazing incidence angle X-ray diffraction (GIAXRD) analyses using CuKα radiation (Rigaku, D/MAX-2500). The microstructures of the ZnON thin films were examined using a transmission electron microscope [(TEM), JEOL, JEM-ARM200F] operated at 200 keV. The scale bar at high magnification was calibrated with the Si (111) plane spacing as a reference. An electron energy-loss spectroscopy [(EELS), Gatan, Enfina 1000] analysis was performed in the Cs-corrected scanning TEM (STEM) mode. The probe size used for the EELS analysis was approximately 0.2 nm. The energy resolution was measured and found to be 1.2 eV in a vacuum in terms of the full width at half maximum of zero loss. EELS spectra were calibrated using the O K edge (532 eV) of SiO_2_ and the Si L edge (99 eV) of the Si substrate. The background was subtracted using a power-law curve-fitting technique. Elemental distributions (line profiles) were extracted based on EELS spectrum imaging (SI) across the films. Hall measurements (Ecopia, HMS-3000) were taken at room temperature to collect information about the Hall mobility and carrier density. Height profile images of the film surfaces were obtained by atomic force microscopy [(AFM), Asylum Research, MFP-3D-BIO]. An X-ray photoelectron spectroscopy [(XPS), Thermo Fisher Scientific Inc., Theta Probe Angle-Resolved X-ray Photoelectron Spectrometer System] analysis was carried out to investigate the bonding states in the films. The sample surfaces were sputtered for 1 min using Ar ions at a sputtering rate of 0.2 nm/sec with reference to SiO_2_ in order to remove any surface contamination. The measured bond energies were referenced to the Zn 2p_3/2_ peak located at 1021.62 eV. Optical spectra were acquired with an ultraviolet-visible-near-infrared spectrophotometer (Shimadzu, SolidSpec-3700).

For the fabrication of the TFT devices, highly doped Si substrates were used as gate electrodes and thermally oxidized SiO_2_ (100 nm) films were used as gate insulators. After sputter-depositing the active layers of ZnON as described above, 100-nm-thick molybdenum (Mo) films were grown by direct-current (DC) sputtering at room temperature using a Mo metal target in a pure Ar plasma to form the source/drain (S/D) electrodes. The active and S/D layers were patterned using shadow masks. The final devices were annealed in air at 250 °C for 1 hr, and their electrical parameters were evaluated using a HP 4145B semiconductor parameter analyzer in a dark room under ambient conditions. The channel width and length of each TFT device were 800 and 200 μm, respectively.

### Computational Details

We performed spin-polarized DFT calculations on a plane-wave basis with the VASP code^42^ and the HSE06 hybrid-functional^38^ with 32% of the HF exchange energy modified to fit the experimental bandgap of Zn_3_N_2_^39^. We chose a primitive unit cell of Zn_3_N_2_ with 24 Zn and 16 N atoms as a reference structure of Zn_3_N_2_. The interaction between the ionic core and the valence electrons was described by the projector augmented wave (PAW) method^43^ and the valence electrons with a plane wave basis up to an energy cutoff of 400 eV. The Brillouin zone was sampled with a 4 × 4 × 4 *k*-points grid. The convergence criteria for the electronic structure and the geometry were 10^−4^ eV and 0.02 eV/Å, respectively. We used the Gaussian smearing method with a finite temperature width of 0.01 eV in order to improve the convergence of states near the Fermi level.

## Additional Information

**How to cite this article**: Park, J. *et al*. A study on the electron transport properties of ZnON semiconductors with respect to the relative anion content. *Sci. Rep*. **6**, 24787; doi: 10.1038/srep24787 (2016).

## Supplementary Material

Supplementary Information

## Figures and Tables

**Table 1:** Electrical parameters of the TFT devices based on films A through E.

Condition	μ_FE_(cm^2^/Vs)	V_th_ (V)	S (V/dec)
A	N/A	N/A	N/A
B	35.9	−9.64	5.09
C	42.6	−6.84	3.61
D	128	−4.24	1.34
E	6.60	−0.65	0.97
