# Supplementary material for: A study on the electron transport properties of ZnON semiconductors with respect to the relative anion content
Source: Sci Rep. 2016 Apr 21;6:24787. doi: 10.1038/srep24787 (PMC4838846; doi:10.1038/srep24787)
Supplement: Supplementary Information [file srep24787-s1.docx]

**Supplementary Information**

**A study on the electron transport properties of ZnON semiconductors with respect to the relative anion content**

**Jozeph Park^1*†^, Yang Soo Kim^2*^, Kyung-Chul Ok^3^, Yun Chang Park^4^, Hyun You Kim^2^, Jin-Seong Park^3^ & Hyun-Suk Kim^2^**

^1^Department of Materials Science and Engineering, Korea Advanced Institute of Science and Technology, Daejeon 305-338, Republic of Korea

^2^Department of Materials Science and Engineering, Chungnam National University, Daejeon 305-764, Republic of Korea

^3^Division of Materials Science and Engineering, Hanyang University, Seoul 133-719, Republic of Korea

^4^National Nano Fab Center, Daejeon 305-806, Republic of Korea

Correspondence and requests for materials should be addressed to H.Y.K. (email: [kimhy@cnu.ac.kr](mailto:kimhy@cnu.ac.kr)) or J.-S.P. (email: [jsparklime@hanyang.ac.kr](mailto:jsparklime@hanyang.ac.kr)) or H.-S.K. (email: [khs3297@cnu.ac.kr](mailto:khs3297@cnu.ac.kr))

^*^These authors contributed equally to this work

^†^Current affiliation: R&D Center, Samsung Display, Yongin-Si, Giheung-gu, Republic of Korea

**S1. AFM topography images of the ZnON films**

Figure S1: Atomic force microscopy (AFM) topography images of the ZnON ﬁlms deposited with different RF sputtering power and oxygen to nitrogen gas flow rate ratios (A: 100 W, 0%, B: 75 W, 1%, C: 50 W, 1%, D: 30 W, 1%, E: 15 W, 4%).

**S2. Low magnification TEM images of the ZnON films**

Figure S2: Low magnification cross-sectional transmission electron microscopy (TEM) images of the ZnON ﬁlms deposited with different RF sputtering power and oxygen to nitrogen gas flow rate ratios (A: 100 W, 0%, B: 75 W, 1%, D: 30 W, 1%, E: 15 W, 4%). In order to avoid oxidation of the ZnON films upon prolonged exposure to air, thin capping layers of ZnO (10 ~ 15 nm) were sputter deposited on top of each film.

**S3. EELS spectra of ZnON**

Figure S3: Electron Energy Loss Spectroscopy (EELS) spectra at various cross-sections of ZnON film (D: 30 W, 1%). Clear EELS signals of zinc (Zn-M), nitrogen (N-K), and oxygen (O-K) are observed.
